# Integrating Long‐Read Nanopore Sequencing for Precision Resolution of Genomic Variants in Dystonia

**DOI:** 10.1002/mds.70072

**Published:** 2025-09-30

**Authors:** Ugo Sorrentino, Martin Pavlov, Nazanin Mirza‐Schreiber, Melanie Brugger, Theresa Brunet, Eugenia Tsoma, Alice Saparov, Ivana Dzinovic, Philip Harrer, Antonia M. Stehr, Matias Wagner, Erik Tilch, Barbara Wallacher, Shiraz Alhasan, Anne Koy, Alessio Di Fonzo, Miriam Kolnikova, Katarina Kusikova, Petra Havrankova, Raushana Tautanova, Sandy Lösecke, Sebastian Eck, Sylvia Boesch, Jan Necpal, Matej Skorvanek, Robert Jech, Holger Prokisch, Juliane Winkelmann, Konrad Oexle, Elisabeth Graf, Michael Zech

**Affiliations:** ^1^ Institute of Human Genetics, School of Medicine and Health Technical University of Munich Munich Germany; ^2^ Institute of Neurogenomics Helmholtz Zentrum München Munich Germany; ^3^ Neurogenetic Systems Analysis Group Institute of Neurogenomics, Helmholtz Munich Neuherberg Germany; ^4^ Bavarian Genomes Network for Rare Disorders Munich Germany; ^5^ Regional Clinical Center of Neurosurgery and Neurology, Department of Family Medicine and Outpatient Care Uzhhorod National University Uzhhorod Ukraine; ^6^ Department of Neurology and Center of Clinical Neuroscience First Faculty of Medicine Charles University and General University Hospital in Prague Prague Czech Republic; ^7^ Division of Pediatric Neurology and Developmental Medicine and LMU Center for Children with Medical Complexity, Dr. von Hauner Children's Hospital, LMU Hospital, Ludwig‐Maximilians‐Universität Munich Germany; ^8^ Specialist Center for Pediatric Neurology, Neurorehabilitation and Epileptology, Schön Clinic Vogtareuth Germany; ^9^ Department of Pediatrics, Faculty of Medicine and University Hospital Cologne University of Cologne Cologne Germany; ^10^ Center for Rare Diseases, Faculty of Medicine and University Hospital Cologne University of Cologne Cologne Germany; ^11^ Dino Ferrari Center, Neuroscience Section, Department of Pathophysiology and Transplantation University of Milan Milan Italy; ^12^ Foundation IRCCS Ca′ Granda Ospedale Maggiore Policlinico, Neurology Unit Milan Italy; ^13^ Department of Pediatric Neurology, Faculty of Medicine, Comenius University University Hospital Bratislava and National Institute of Children's Diseases Bratislava Slovakia; ^14^ Department of Neurosurgery, Medical Centre Hospital of the President's Affairs Administration of the Republic of Kazakhstan Astana Kazakhstan; ^15^ Department of Neurology Medical University of Innsbruck Innsbruk Austria; ^16^ Department of Neurology, Zvolen Hospital Zvolen Slovakia; ^17^ Parkinsonism and Movement Disorders Treatment Center, Zvolen Hospital Zvolen Slovakia; ^18^ Department of Neurology P. J. Safarik University Kosice Slovakia; ^19^ Department of Neurology University Hospital of L. Pasteur Kosice Slovakia; ^20^ Deutsches Zentrum Für Kinder‐ und Jugendgesundheit Munich Germany; ^21^ Deutsches Zentrum Für Psychische Gesundheit Munich Germany; ^22^ Munich Cluster for Systems Neurology, SyNergy Munich Germany; ^23^ Institute for Advanced Study Technical University of Munich Garching Germany

**Keywords:** long‐read sequencing, dystonia, long‐range phasing, complex structural variants, repeat expansions, nanopore technology

## Abstract

**Background:**

Although many individuals with dystonia present with features indicative of single‐gene etiologies, obtaining definitive genetic diagnoses can be challenging.

**Objective:**

We assessed the value of nanopore‐based long‐read sequencing (LRS) in achieving molecular clarification of dystonic syndromes.

**Methods:**

From a large dystonia cohort with short‐read sequencing (SRS) data, 14 cases with unclear, difficult‐to‐evaluate, or missing causative variants were recruited. Long‐read whole‐genome sequencing was performed according to Oxford Nanopore Technologies (ONT) protocols.

**Results:**

ONT sequencing produced long‐range haplotypes, variant calls inaccessible to short‐read technology, as well as methylation data. Phase inference allowed for changes in variant classification, establishing compound heterozygosity of causative variants in four cases. We illustrate an important advantage of LRS compared with SRS in (re)defining the identity of dystonia‐causing structural variants and repeat expansions for seven individuals. One patient was found to harbor a novel exonic LINE‐1 insertion in *SGCE*, expanding the genetic mechanism in myoclonus‐dystonia. ONT data also provided unexpected insights into apparent mosaic expanded repeats in *FMR1* in a subject with isolated focal dystonia. We further showed that LRS outperformed SRS in avoiding erroneous calls resulting from confounding pseudogene sequences and in discovering pathogenic alterations missed by conventional pipeline utilization (three cases). Moreover, simultaneous methylome analysis aided in directing the interpretation of three variants, including a *KMT2B* variant of uncertain significance that was reclassified as causal by LRS‐based episignature profiling.

**Conclusions:**

ONT‐based LRS uniquely improves analysis of dystonia‐associated variations that had not previously been resolved by SRS, implying broad utility for future exploration of the molecular origins of the condition. © 2025 The Author(s). *Movement Disorders* published by Wiley Periodicals LLC on behalf of International Parkinson and Movement Disorder Society.

Current molecular testing majorly relies on high‐throughput methods based on short‐read sequencing (SRS).^1^ The introduction of exome sequencing (ES) and short‐read genome sequencing (srGS) in clinical practice simplified workflows, made them more effective, and increased diagnostic yield, especially in case of genetically heterogeneous conditions.^1^ Nevertheless, technological progress has not overcome a number of intrinsic limitations of SRS.^2^, ^3^ These include lack of long‐range haplotype information and insufficient resolution of structural variants (SVs), including complex SVs (cxSVs).^2^, ^3^ Moreover, comprehensive characterization of tandem repeat expansions (TREs) and the investigation of single‐nucleotide variants (SNVs) and short insertions/deletions (indels) in regions with high sequence homology or other complex structural features remain challenging in SRS‐based diagnostic settings.^2^, ^3^ As a result, other dedicated analysis strategies are becoming increasingly popular, such as methods that provide long‐read sequencing (LRS) capabilities.^4^, ^5^, ^6^ LRS allows for the study of much larger genomic fragments compared with earlier ES/srGS approaches.^6^ Long‐read technologies can outperform alignment‐based SRS in genome‐assembly quality and genome‐wide discovery of difficult‐to‐map, clinically relevant variants.^6^ In addition, new applications enabling parallel assessment of DNA sequence and DNA modification data render LRS a potential gold standard for evaluation of hereditary diseases that impact on methylation.^7^ Although a growing body of literature supports the usefulness of LRS for the elucidation of hidden causes of rare‐disease manifestations,^8^, ^9^, ^10^ the role of this method has not yet been examined systematically in dystonia.^11^ Whether LRS can serve as a high‐quality alternative with complementary strengths for diagnostic evaluation of dystonia‐affected individuals who present with different types of unresolved or inconclusive genomic findings is unclear. Indeed, the molecular basis of dystonic disorders is extremely heterogeneous,^12^ comprising a broad spectrum of genomic alterations that often go undetected with standard sequencing technologies.^13^

In this study, we aimed to assess the power of LRS for phasing, resolving, and discovering variants in patients with dystonia. We report the detailed investigation of 14 diagnostically indeterminate priority cases from a large cohort, in whom Oxford Nanopore Technologies (ONT, Oxford, UK)‐based long‐read genome sequencing (ONT lrGS) was instrumental for accurate follow‐up exploration, leading to an increased rate of diagnosis and novel insights into challenging dystonia‐related variation.

## Subjects and Methods

### Studied Patients

**FIG. 1:**
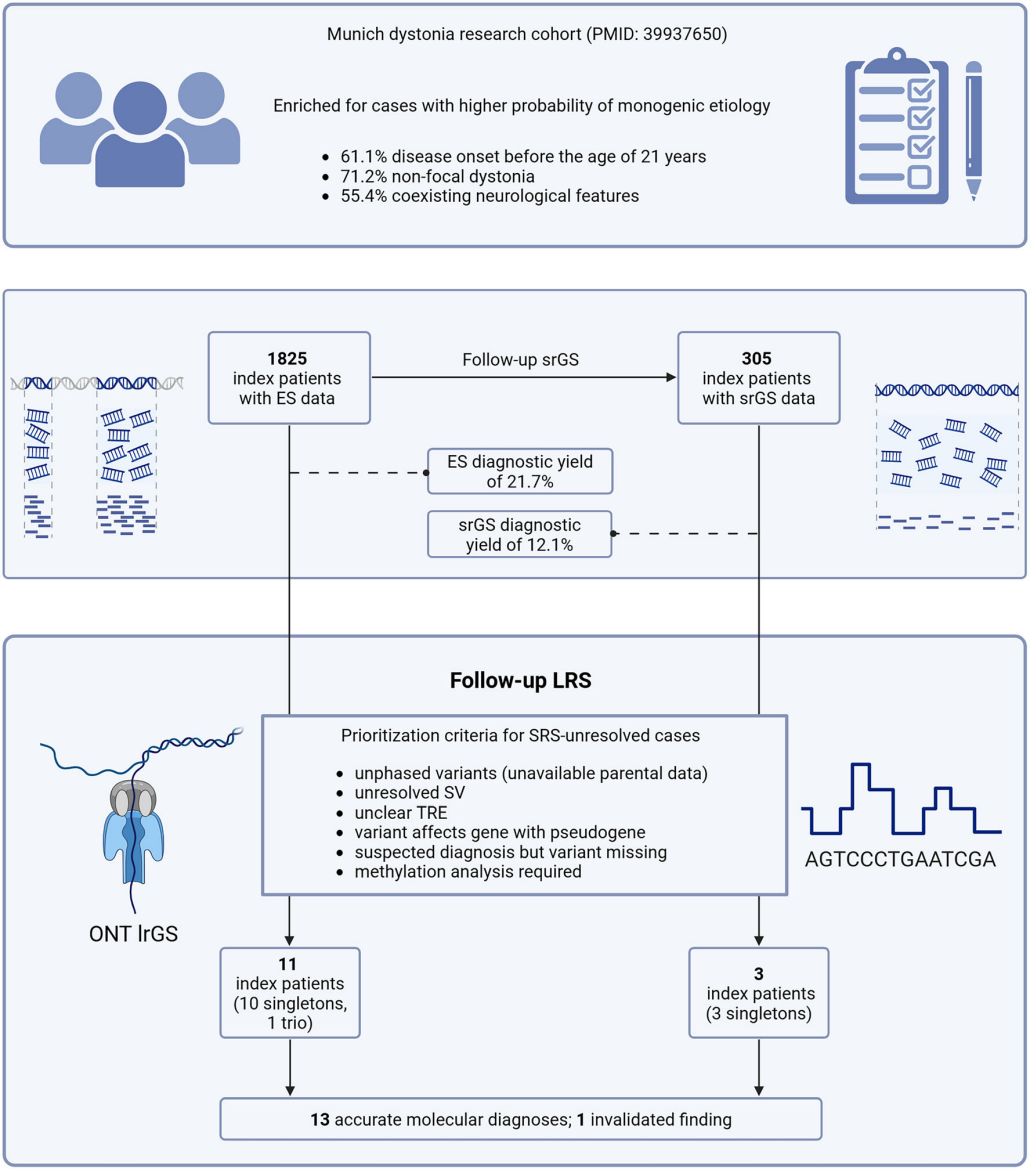
Overview of the dystonia research pipeline and the design of the LRS follow‐up. Results for our unique cohort of 1825 index patients with different forms of dystonia were previously reported after accomplishment of ES (n = 1825) and srGS (n = 305).^13^, ^14^ A total of 21.7% of the index patients were diagnosed by ES, whereas another 12.1% of the patients received a diagnosis after completion of srGS.^13^, ^14^ For follow‐up ONT lrGS, we prospectively included 14 expert‐selected index patients who were found by SRS to carry unresolved, potentially clinically relevant variations or who were considered by movement disorder specialists at the recruiting site to have a certain clinical disorder for which no corresponding diagnostic genotype was identified. Eligibility criteria for the ONT lrGS study arm are specified. The sources and numbers of the LRS‐analyzed cases are shown, along with a summary of the outcome. ES, exome sequencing; LRS, long‐read sequencing; ONT lrGS, Oxford Nanopore Technologies–based long‐read genome sequencing; srGS, short‐read genome sequencing; SRS, short‐read sequencing. Created with bioRender (https://www.biorender.com). [Color figure can be viewed at wileyonlinelibrary.com]

The patients selected for in‐depth evaluation by ONT lrGS were part of a genomic sequencing research program for dystonic diseases (Munich, Germany),^13^, ^14^ which included 1825 index patients (52.3% female; as of January 2025).^13^ Of these individuals, 61.1% had disease onset before the age of 21 years, 71.2% had nonfocal dystonia, and 55.4% had coexisting neurological features.^13^ All index patients had undergone ES.^13^, ^14^ In addition, we recently conducted a genome analysis flagship study where we applied srGS to 305 ES‐negative cases.^13^ A total of 78.3% (1429/1825) of the patients remained without a conclusive genetic diagnosis after ES, whereas 87.9% (268/305) of the srGS‐analyzed patients had no finding with an appropriate degree of diagnostic certainty.^13^ One goal of our program is to build a resource for benchmarking of new analytic technologies, thereby increasing the etiological yield and providing new insights into causative mutational mechanisms. Given the advantages of LRS,^11^ we pursued ONT lrGS in a subset of participating cases with candidate aberrations from our ES/srGS analyses that we have not been able to confidently interpret. We also enrolled patients with “missing”^15^ pathogenic variants to evaluate whether ONT lrGS can be effectively used to establish a molecular diagnosis in these cases. To that end, we recruited samples expected to benefit from LRS according to the following criteria: (1) patient had two prioritized variants in a recessive disease gene, but it was unknown whether both alleles were affected; (2) patient was suspected to harbor an SV of clinical relevance, but (sufficient) resolution could not be achieved; (3) patient was identified with an imprecisely called TRE that was roughly estimated to be longer than the disease threshold; (4) patient carried a suspicious rare variant in a disease gene for which a pseudogene with high sequence similarity was known^16^; and (5) patient was considered as having a high probability of a certain rare‐disease diagnosis on the basis of clinical signs^17^ or preliminary genetic evidence, but a causative variation in the gene of interest could not be unambiguously identified by SRS.^18^ Finally, we explored the capacity of our ONT assay to simultaneously assess DNA methylation for different indications in a proof‐of‐concept approach. An overview of the study workflow with participant selection is outlined in Figure 1. The study conformed to the Declaration of Helsinki principles, and all subjects and/or their caregivers provided written informed consent. Ethics review board approval was obtained from the involved centers.

### SRS and Inconclusive Genomic Constellations

The 14 included patient samples had been subjected to prior ES, and three samples were also analyzed by srGS (Fig. 1).^13^ ES and srGS were carried out using established Illumina sequencing protocols, and the methodologies for SRS variant calling and interpretation have been detailed elsewhere.^13^, ^14^ SNVs and indels were identified with custom SRS pipelines based on GATK and Samtools.^13^, ^14^ ExomeDepth was employed for the detection of copy‐number variants (CNVs) in ES data,^19^ whereas ExpansionHunter was used for TRE discovery from srGS.^20^ Singleton ES prioritized unphased variants in *PNPT1* (patient 1), *SZT2* (patient 2), *TH* (patient 3), and *HIBCH* (patient 4); hard‐to‐resolve SV structures in *MECP2* (patients 5 and 6) and *SGCE* (patient 7); and a candidate alteration in *PI4KA* (patient 11), a gene that has two known pseudogenes.^21^ Missing disease‐associated variation in trio ES data was deemed likely for two cases (patient 12, *FOXG1*; patient 13, *OTUD7A*), whereas another individual (patient 14) had a singleton ES‐identified variant of uncertain significance (VUS)^22^ in *KMT2B* that required methylation analysis. Potential outlier TREs^20^ were found in singleton srGS datasets of three participants (patient 8, *TBP*; patient 9, *FMR1*; patient 10, *DIP2B*). In patients 1 to 4, variant phases were undeterminable upon SRS because the parents and/or other relatives were not available for testing because the patient was a refugee with no contact with family or lived in a foster home, or the patient's parents and/or relatives were deceased, or other reasons were present.

### ONT lrGS

We chose to sequence the entire genome of each study participant (13 singletons, 1 trio) using an ONT approach.^11^ DNA was extracted from blood using either the Prepito DNA Blood400 Kit (Revvity) or the Nanobind CBB Kit (PacBio); 1500 ng of DNA was fragmented to a target size of 25 kb using Megaruptor 3 (Diagenode, Liége, Belgium). Whole‐genome libraries were prepared from 1000 ng of DNA according to ONT's Ligation Sequencing Kit V14 protocol. After purification, each library was sequenced over a total sequencing period of 72 h on a PromethION 24 device using ONT R10.4.1 flow cell chemistry. For base calling, we employed the super‐accurate basecalling model (v4.3.0) following ONT‐recommended guidelines.

### Assessment of ONT lrGS Data

Bioinformatics processing and downstream analyses of the ONT lrGS data were performed by applying the automated open‐source “Epi2me‐labs/ wf‐human‐variation” (v2.3.0) workflow (https://github.com/epi2me-labs/wf-human-variation) provided by ONT,^11^ which used minimap2 v2.28‐r1209 for read alignment against the human reference genome (GRCh38/hg38; GenBank assembly GCA_000001405.15). Details of variant analysis can be found in Supporting Information Methods.

## Results

We successfully undertook ONT lrGS in 16 individuals encompassing one dystonia trio (patient plus unaffected parents) and 13 singleton dystonia patients (Fig. 1). Across these samples, a median data output of 129.6 Gb and a median N50 of 6513.5 bp were obtained. We produced a median of 26.7 × 10^6^ total reads per sample. With this yield, we achieved a median genome‐wide coverage of 41.8×. Of the 19 assessed variations (Table 1), 11 (57.9%) were SNVs/indels, 5 (26.3%) were SVs, and 3 (15.8%) were TREs. Using the relevant variant‐calling algorithms and direct visual investigation,^23^ we were able to establish accurate diagnoses for 13 patients and to invalidate a potentially diagnostic finding from SRS in another individual (Table 1).

**TABLE 1:** Summary of patients and variants investigated by ONT lrGS in this study

Participant (sex, age [y])	Selected phenotype features	LRS inclusion category	Previous SRS test	Previous ES/srGS result	ONT lrGS result; genotype annotation	Genotype interpretation (PMIDs: 25741868, 31690835, 36507974); final diagnosis (OMIM)
Patient 1 (F, 19)	Dystonia, developmental delay	Phasing of variants^a^	Singleton ES	Unphased *PNPT1* variants (SNV/SNV)	Compound heterozygous *PNPT1* variants; NM_033109.5:c.[1519G>T];[1906+1G>T]	P + LP; combined oxidative phosphorylation deficiency 13 (MIM: 614932)
Patient 2 (F, 36)	Dystonia, developmental arrest, epilepsy, dysmorphia	Phasing of variants^a^	Singleton ES	Unphased *SZT2* variants (indel/SNV)	Compound heterozygous *SZT2* variants; NM_001365999.1:c.[5961del];[6284A>G]	LP + LP; developmental and epileptic encephalopathy 18 (MIM: 615476)
Patient 3 (M, 2)	Dystonia, myoclonus, tremor, hypotonia	Phasing of variants^a^	Singleton ES	Unphased *TH* variants (SNV/SNV)	Compound heterozygous *TH* variants; NM_000360.4:c.[635A>C];[1282C>T]	LP + P; Segawa syndrome, recessive (MIM: 605407)
Patient 4 (M, 19)	Dystonia, developmental delay, intellectual disability, MRI abnormalities	Phasing of variants^a^	Singleton ES	Unphased *HIBCH* variants (CNV/SNV)	Compound heterozygous *HIBCH* variants, 1 variant characterized as cxSV event (deletion, insertion); NC_000002.12:g.[190247591_190249868del190242687_190242718ins];[190244946C>T]	P + LP; 3‐hydroxyisobutryl‐CoA hydrolase deficiency (MIM: 250620)
Patient 5 (F, 5)	Dystonia, stereotypies, developmental stagnation, epilepsy	SV analysis	Singleton ES	Unresolved SV in *MECP2* (possible cxSV event)	Heterozygous cxSV involving *MECP2* exons 2 and 3 (deletion, inverted duplication); NC_000023.11:g.154030711_154030862delins154030370_154034129inv	P; Rett syndrome (MIM: 312750)
Patient 6 (F, 14)	Dystonia, speech impairment, epilepsy	SV analysis	Singleton ES	Unresolved SV or larger indel in *MECP2* (possible deletion within exon 3)	Heterozygous intra‐exonic *MECP2* deletion CNV (exon 3); NM_001110792.2:c.1188_1239del	P; Rett syndrome (MIM: 312750)
Patient 7 (F, 54)	Dystonia, myoclonus	SV analysis	Singleton ES	Unresolved SV in *SGCE* (possible exon 6 deletion)	Heterozygous truncated LINE‐1 structure affecting *SGCE* exon 6; NM_003919.3:c.793_794ins[NC_000023.11:g.11712154_11713316]	P; dystonia‐11, myoclonic (MIM: 159900)
Patient 8 (F, 44)	Dystonia, parkinsonism, behavioral disturbances	TRE analysis	Singleton srGS	Potential outlier TRE in *TBP* (reduced‐penetrance range)	Heterozygous exonic *TBP* TRE in full‐penetrance range (53 CAG‐CAA repeats); NM_003194.5:c.172_285CAG[3]CAA[3]CAG[6]CAA[1]CAG[1]CAA[1]CAG[36]CAA[1]CAG [1]	Pathological range (PMID: 34034831); spinocerebellar ataxia 17 (MIM: 607136)
Patient 9 (F, 35)	Dystonia	TRE analysis	Singleton srGS	Potential outlier TRE in *FMR1* (premutation range)	Heterozygous 5′ UTR *FMR1* TRE with suspected mosaicism (premutation and full mutation), hypermethylated full‐mutation–containing reads; NM_002024.6:c.‐128_‐69GGM[123_153]//−128_‐69GGM[217_500]	Pathological range (PMID: 34034831); fragile X–associated tremor/ataxia syndrome (MIM: 300623)
Patient 10 (M, 38)	Dystonia, history of mixed hyperkinetic movements during childhood	TRE analysis	Singleton srGS	Potential outlier TRE in *DIP2B*	Heterozygous 5′ UTR *DIP2B* TRE in pathological range (PMID: 39854091), hypermethylation of read with large expansion; NM_173602.3:c.‐139_‐119GGC[186_725]	Pathological range (PMID: 34034831); *DIP2B*‐related movement disorder (PMID: 39854091)
Patient 11 (M, 9)	Dystonia, spasticity, developmental delay, MRI abnormalities	Gene‐pseudogene pair analysis	Singleton ES	Homozygous *PI4KA* candidate variant call (SNV)	Homozygous status of *PI4KA* variant invalidated; heterozygous variant NM_058004.4:c.5903A>G	N/A; inconclusive
Patient 12 (F, 14)	Dystonia, developmental delay, intellectual disability	Missing variant	Trio ES	Apparently homozygous *OTUD7A* variant (SNV) with inconclusive parental segregation	“Hemizygous” *OTUD7A* SNV in *trans* with heterozygous ~1.65‐Mb *OTUD7A*‐containing deletion CNV; NC_000015.10:g.[(30585641_30604124)_(32225000_32260026)del];[31503820 T>C]	P + P; neurodevelopmental disorder with hypotonia and seizures (MIM: 620790)
Patient 13 (F, 3)	Dystonia, myoclonus, developmental delay, microcephaly	Missing variant	Trio ES	No variant in clinically suspected candidates	Heterozygous *FOXG1* indel affecting GC‐rich region with no coverage in ES data; NM_005249.5:c.256dup	P; Rett syndrome, congenital variant (MIM: 613454)
Patient 14 (F, 61)	Dystonia	Parallel genomic‐variant and methylation analysis	Singleton ES	VUS (in‐frame indel) in *KMT2B*	Heterozygous *KMT2B* indel, *KMT2B*‐typical methylation pattern in blood (PMID: 34590685); NM_014727.3:c.8065_8070del	LP; dystonia 28, childhood onset (MIM: 617284)

*Note*: Coordinates provided for ONT lrGS results refer to GRCh38/hg38. HGVS nomenclature is given where appropriate.

Abbreviations: ONT lrGS, Oxford Nanopore Technologies–based long‐read genome sequencing; LRS, long‐read sequencing; SRS, short‐read sequencing; ES, exome sequencing; srGS, short‐read genome sequencing; OMIM, Online Mendelian Inheritance in Man; F, female; P, pathogenic; LP, likely pathogenic; M, male; SNV, single‐nucleotide variant; indel, short insertion/deletion; MRI, magnetic resonance imaging; CNV, copy‐number variant; CoA, coenzyme A; SV, structural variant; cxSV, complex structural variant; LINE‐1, long interspersed nuclear element‐1; TRE, tandem repeat expansion; N/A, not applicable; VUS, variant of uncertain significance; HGVS, Human Genome Variation Society.

^a^ Phase undeterminable upon SRS analysis because parents/relatives were not available for testing: patient was refugee with no contact to family or lived in foster home, or patient's parents/relatives were deceased, or other reason.

### Phasing of Variants

**FIG. 2:**
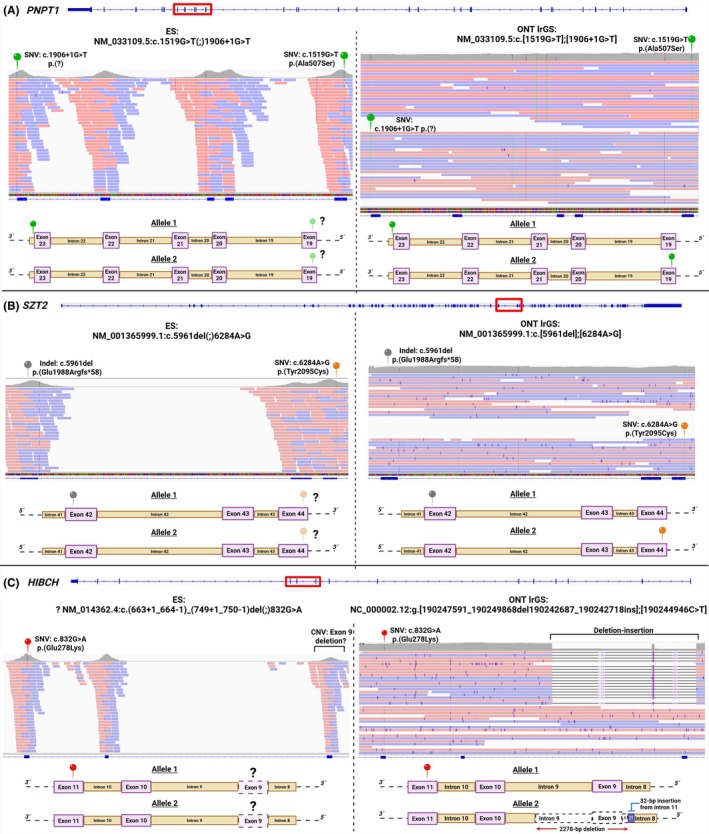
Examples of successful variant phasing performed on patients 1, 2, and 4. Visualizations of genomic alignments of previously acquired ES data^13^, ^14^ and herein‐produced ONT lrGS data in the IGV software^23^ are shown. ES variant annotations were based on genome assembly GRCh37/hg19,^13^, ^14^ whereas coordinates provided for ONT lrGS data refer to GRCh38/hg38. The canonical transcript (or relevant parts of the transcript) of each analyzed gene are illustrated at the top of each comparison between ES and ONT lrGS outcomes. IGV zoom‐in regions highlight the called variants, which are described according to HGVS nomenclature. Schematic representations of the allelic configurations before and after resolution by LRS are depicted. Alignments are phased into separate haplotypes to determine that the two variants were in *trans*, establishing compound heterozygous diagnoses related to *PNPT1* in patient 1 (**A**), *SZT2* in patient 2 (**B**), and *HIBCH* in patient 4 (**C**). Note the more complex nature of the *HIBCH*‐disrupting SV in ONT lrGS data compared with the ES result (allele 2 in **C**). CNV, copy‐number variant; ES, exome sequencing; HGVS, Human Genome Variation Society; IGV, Integrative Genomics Viewer; indel, short insertion/deletion; LRS, long‐read sequencing; ONT lrGS, Oxford Nanopore Technologies–based long‐read genome sequencing; SNV, single‐nucleotide variant. [Color figure can be viewed at wileyonlinelibrary.com]

We used ONT lrGS to produce phased haplotypes, identifying compound heterozygosity in four singleton cases. The eight responsible variants, encompassing six SNVs, one indel, and one SV, had escaped conventional phase determination because parental sequencing data were unavailable (Table 1). In the first individual (patient 1) affected by developmental delay and limb dystonia, we phased two ES‐identified heterozygous *PNPT1* SNVs into separate haplotypes, confirming their biallelic status and securing the diagnosis^22^ of combined oxidative phosphorylation deficiency‐13 (MIM: 614932) (Table 1; Fig. 2A). Patient 2 with developmental arrest, epilepsy, and generalized dystonia was found by ES to carry a heterozygous, likely pathogenic frameshift indel and a heterozygous missense VUS in *SZT2*, the gene linked to developmental and epileptic encephalopathy‐18 (MIM: 615476). By generating reads spanning both mutant positions, ONT lrGS demonstrated that the variants were in *trans*, thus resolving the VUS^22^ (Table 1; Fig. 2B). Patient 3 manifested multiple types of movement disorders including prominent dystonia and was found by ES to bear two heterozygous SNVs, a known pathogenic nonsense alteration and a described^24^ missense variant, in *TH*. ONT‐based variant phasing indicated compound heterozygosity, firmly establishing the cause of the patient's condition^22^ (autosomal recessive Segawa syndrome; MIM: 605407; Table 1; Fig. S1). Moreover, patient 4, an individual with early‐onset dystonia and comorbid neurodevelopmental impairment, required LRS to achieve resolution of the underlying genetic pathology. ES reported the presence of a heterozygous missense VUS and a potential, heterozygous single‐exon deletion CNV in *HIBCH*. ONT lrGS not only showed that both alleles were mutated but also highlighted unexpected new information on the exact configuration of the latter variation (Table 1; Fig. 2C). Instead of a “simple” exon 9 loss (NM_014362.4) suggested by SRS, visualization of the region indicated a combination of deleted and inserted sequences, with a range of SV sizes from 32 bp (insertion) to 2278 bp (deletion encompassing the affected exon; Fig. 2C). Follow‐up review of patient 4's brain MRI showed abnormal signal intensities in the basal ganglia bilaterally, supporting the diagnosis of 3‐hydroxyisobutyryl‐CoA hydrolase deficiency (MIM: 250620). We (re)classified the SV event and the missense variant as pathogenic and likely pathogenic,^25^, ^26^ respectively.

### Structural Variants

**FIG. 3:**
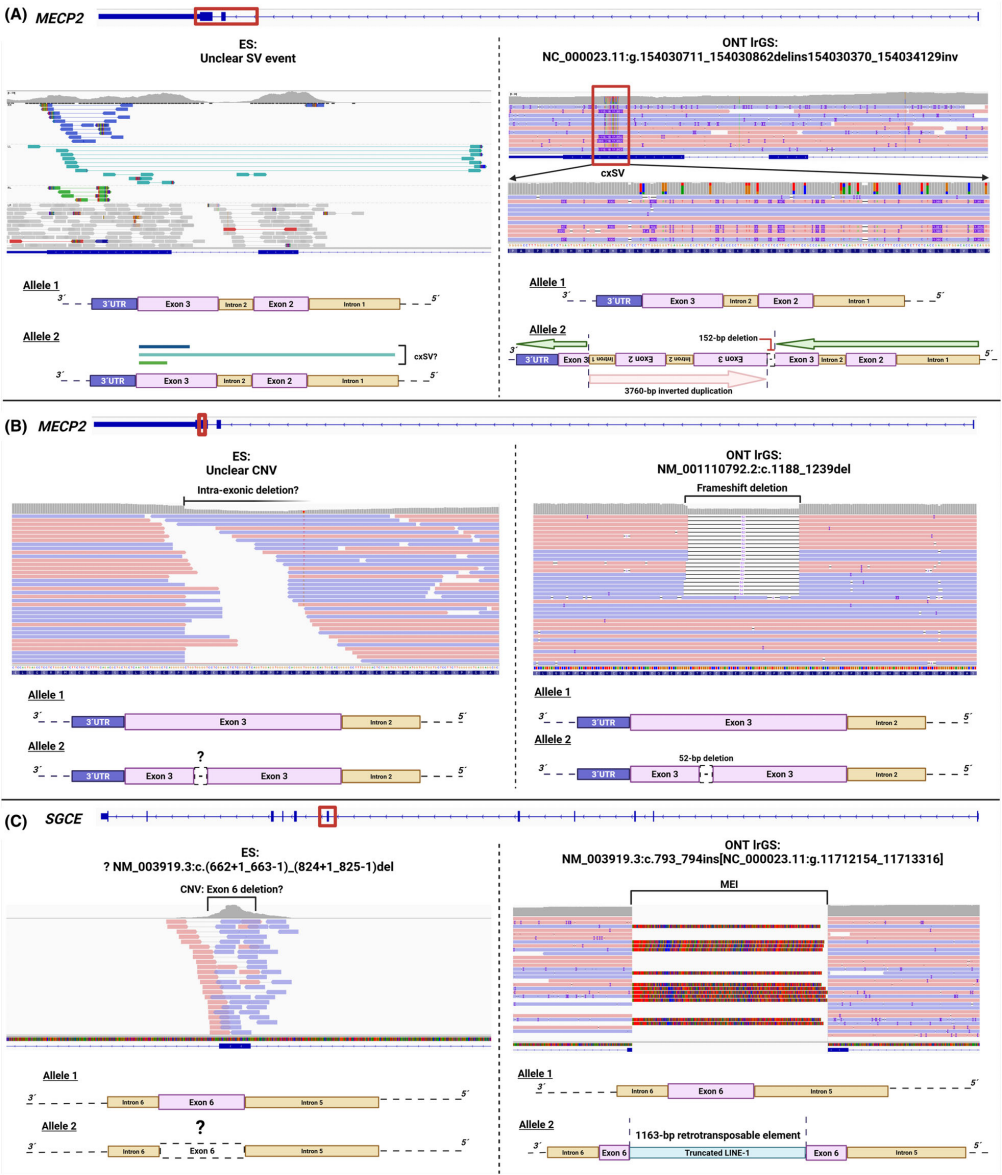
Precise characterization of SV events in patients 5, 6, and 7. Visualizations of genomic alignments of previously acquired ES data^13^, ^14^ and herein‐produced ONT lrGS data in the IGV software^23^ are shown. ES variant annotations were based on genome assembly GRCh37/hg19,^13^, ^14^ whereas coordinates provided for ONT lrGS data refer to GRCh38/hg38. The canonical transcript (or relevant parts of the transcript) of each analyzed gene are illustrated at the top of each comparison between ES and ONT lrGS outcomes. IGV zoom‐in regions highlight the unclear and the resolved SVs. HGVS nomenclature is given where appropriate. Schematic representations of the allelic configurations before and after resolution by LRS are depicted. (**A**) In patient 5, ONT lrGS enabled the resolution of a cxSV affecting *MECP2*, whose identity had remained unclear in ES data evaluation including ExomeDepth^19^ analysis. (**B**) In patient 6, an ambiguous CNV in *MECP2* found on closer manual inspection of ES data was characterized by ONT lrGS as a pathogenic frameshift deletion. (**C**) In patient 7, ONT lrGS‐based SV analysis demonstrated the absence of a single‐exon deletion in *SGCE* suggested by ES (ExomeDepth^19^ call). Unexpectedly, a mobile element insertion (truncated LINE‐1 sequence) was present. CNV, copy‐number variant; cxSV, complex structural variant; ES, exome sequencing; HGVS, Human Genome Variation Society; IGV, Integrative Genomics Viewer; LINE‐1, long interspersed nuclear element‐1; LRS, long‐read sequencing; MEI, mobile element insertion; ONT lrGS, Oxford Nanopore Technologies–based long‐read genome sequencing; SV, structural variant; UTR, untranslated region. [Color figure can be viewed at wileyonlinelibrary.com]

ONT lrGS facilitated the unambiguous characterization of uncertain SV events by uncovering their identity, breakpoints, and orientation. Close scrutiny of ONT‐read alignments delineated a cxSV, a small‐length deletion CNV, and the insertion of a long interspersed nuclear element‐1 (LINE‐1) fragment, ranging in size from ~50bp to ~3.8 kb (Table 1). An example of how ONT lrGS together with appropriate SV calling enabled the discovery of a complex rearranged genome structure and predicted the associated breakpoints' locations is provided by the case of patient 5. This female individual had a working diagnosis of Rett syndrome (MIM: 312750), made on the basis of a typical clinical course with developmental stagnation and emergence of mixed movement abnormalities,^27^ including stereotypies and truncal dystonia. ES suggested a possible cxSV event in *MECP2*, but the resolution was not sufficient to formulate a definitive molecular diagnosis (Fig. 3A). Visual inspection of ONT data showed a complex, *MECP2*‐disrupting rearrangement in the heterozygous state, which involved breakpoints within the last exon (NM_001110792.2: exon 3). This cxSV configuration consisted of a 152‐bp deletion in exon 3 (hg38: NC_000023.11:g.154030711_154030862) combined with an inverted duplicated segment encompassing full or partial sequences of exons 2 and 3, as well as introns 1 and 2 (3760 bp; Table 1; Fig. 3A). Investigation of the parents' ONT lrGS data indicated that the cxSV had arisen de novo. Further phasing analysis using informative SNVs highlighted that the de novo cxSV had occurred on the paternal X chromosome (data not shown). We considered the variation explanatory for the Rett syndrome phenotype,^25^, ^26^ ending the diagnostic journey. The second case in whom ONT lrGS refined SV interpretation involved patient 6, who was also clinically suspected to have Rett syndrome. This female individual presented regression in language skills, generalized dystonia, and epilepsy. Although analysis using our ES pipeline did not automatically detect an *MECP2* alteration, a potential CNV affecting the gene's mutational hot spot region^28^, ^29^ was apparent in the SRS data on manual review (Fig. 3B). The length and consequence of this inaccurately called variant were unclear. ONT lrGS confirmed a small heterozygous intraexonic *MECP2* CNV and delineated the breakpoints of the 52‐bp deletion (Table 1; Fig. 3B). Because the CNV was expected to introduce a frameshift, we subsequently reported it as pathogenic.^25^, ^26^ We clarified uncertainties in the nature of another suspected SV, predicted to be present in patient 7 with dystonia and myoclonus. ES‐based CNV analysis called only one copy of exon 6 of the dystonia‐linked gene *SGCE* (NM_003919.3). Because calls of heterozygous single‐exon deletions are often false positive in ES data,^1^, ^30^ we scrutinized the finding by ONT lrGS. Surprisingly, we found that *SGCE* exon 6 was not disrupted by a deletion but by an insertion of 1163 bp (Fig. 3C). Comparison of the insertion with consensus sequences using the BLAT alignment tool from the University of California, Santa Cruz (UCSC)‐Genome Browser (https://genome.ucsc.edu/) characterized the variation as a truncated LINE‐1 originating from chromosome X (hg38: NC_000023.11:g.11712154_11713316; Table 1; Fig. 3C), highlighting a new causative mechanism^25^, ^26^ for *SGCE*‐related myoclonus‐dystonia (MIM: 159900).

### Tandem Repeat Expansions

**FIG. 4:**
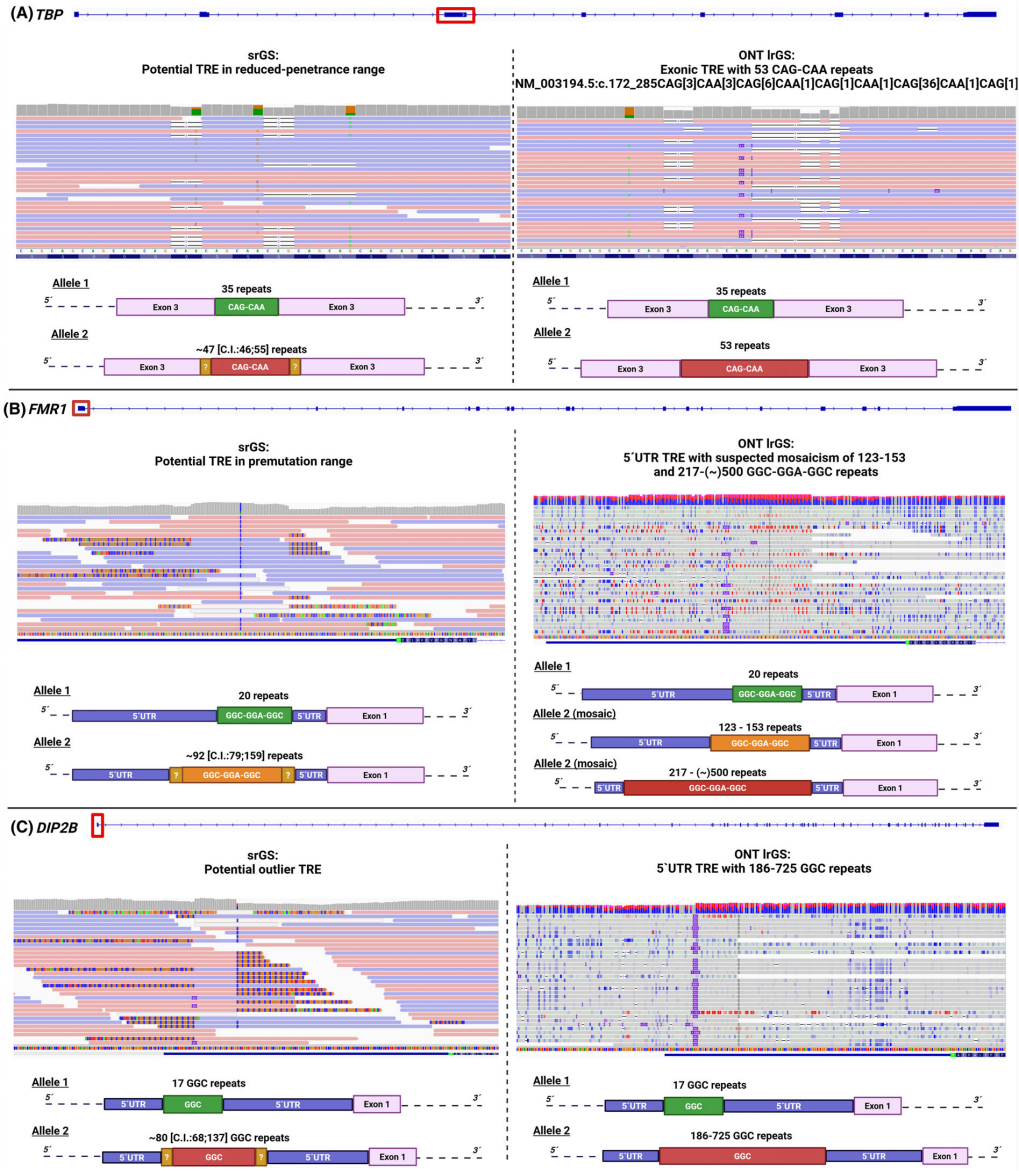
Conclusive TRE analysis enabled for patients 8 to 10. Visualizations of genomic alignments of previously acquired srGS data^13^ and herein‐produced ONT lrGS data in the IGV software^23^ are shown. TREs in srGS data were determined with ExpansionHunter^20^ using the genome assembly GRCh37/hg19,^13^ whereas ONT lrGS‐based TRE analysis was done with direct inspection based on GRCh38/hg38. The canonical transcript (or relevant parts of the transcript) of each analyzed gene are illustrated at the top of each comparison between ES and ONT lrGS outcomes. IGV zoom‐in regions highlight the potential and the confirmed TREs. Schematic representations of the allelic configurations before and after resolution by LRS are depicted. (**A**) In patient 8, ONT lrGS accurately identified the length of a disease‐causing TRE in *TBP*; HGVS nomenclature is given. (**B**) In patient 9, a suspected mosaic constellation of expanded repeat units was uncovered by ONT lrGS for *FMR1*, unidentifiable in prior ExpansionHunter^20^ screening. *FMR1* methylation is marked in red. Computational assembly of phased haplotypes was impaired in this case because of the different sizes of expanded repeats, compatible with mosaicism; a clustering of reads carrying the TREs is illustrated in Figure S6. Following HGVS recommendations, we annotated the *FMR1* repeat as GGC‐GGA‐GGC (https://hgvs‐nomenclature.org). (**C**) In patient 10, ONT lrGS‐based repeat profiling confidently detected a pathological TRE in *DIP2B*, previously linked to dystonic movement disorder phenotypes in an independent family.^34^ *DIP2B* methylation is marked in red. Following HGVS recommendations, we annotated the *DIP2B* repeat as GGC (https://hgvs‐nomenclature.org). C.I., confidence interval (ExpansionHunter); ES, exome sequencing; HGVS, Human Genome Variation Society; IGV, Integrative Genomics Viewer; LRS, long‐read sequencing; ONT lrGS, Oxford Nanopore Technologies–based long‐read genome sequencing; srGS, short‐read genome sequencing; TRE, tandem repeat expansion; UTR, untranslated region. [Color figure can be viewed at wileyonlinelibrary.com]

We examined the potential of ONT lrGS to overcome ambiguities encountered in the analysis of dystonia‐related TREs with SRS. By exploring three potential repeat‐sized alleles that we previously identified using typical ExpansionHunter‐based TRE screening,^13^, ^20^ we generated much more accurate estimates on the expansion lengths and their variability. In patient 8, ExpansionHunter predicted an expanded CAG/CAA repeat in exon 3 of *TBP* (NM_003194.5), associated with spinocerebellar ataxia‐17 (MIM: 607136). Although the potential disruption of this gene was in line with the patient's presentation, comprising adult‐onset dystonia‐parkinsonism and behavioral disturbances, we were unable to confidently diagnose the case because the estimated repeat size corresponded to the reduced penetrance range (~47 repeat units; threshold for full penetrance: >48 units) (Fig. 4A). The analysis of ONT data highlighted a bona fide pathogenic^31^ heterozygous *TBP* expansion with 53 repeats (Table 1; Fig. 4A). We assessed TRE variation in the 5′ untranslated region (UTR) of *FMR1* in another case (patient 9), associated with fragile X syndrome (>200 repeat units; MIM: 300624) or fragile X–associated tremor/ataxia syndrome (FXTAS; 55–200 repeat units; MIM: 300623). This female individual had an ExpansionHunter‐predicted *FMR1* repeat expansion with ~92 units and presented adult‐onset focal isolated dystonia, an unusual but recently reported manifestation of FXTAS.^32^, ^33^ When inspecting region‐spanning ONT reads with high‐quality resolution, a pattern of mosaicism was suspected,^15^ because there existed calls with both premutation‐sized repeats (~120–150 units) and full‐mutation repeat sizes (~200–500 units) (Table 1; Fig. 4B); simultaneous haplotype‐resolved methylation visualization confirmed the presence of repeats with >200 units (hypermethylated calls; Fig. 4B). Based on these results, we diagnosed a mosaic *FMR1* TRE constellation and attributed the patient's dystonic phenotype to the confirmed premutation.^31^, ^32^ Furthermore, ONT lrGS unraveled a likely underidentified TRE‐linked condition^34^ in patient 10 with a presentation of progressive combined dystonia. ExpansionHunter analysis reported an ambiguously mapped repeat expansion in the 5′ UTR of *DIP2B*, a locus linked to neurodevelopmental disease (MIM: 136630). Although *DIP2B* has recently been implicated in movement disorders,^34^ the gene's repeat sizes appear to be significantly underestimated in SRS data,^34^ making it difficult to identify and judge this pathology. Detailed analysis using ONT‐read alignments and methylation profiling showed pathologically expanded *DIP2B* repeats of varying size (>180 units), along with apparent hypermethylation of an allele bearing a very large expansion (Table 1; Fig. 4C). We regarded the finding as diagnostic‐grade evidence,^31^, ^34^ corroborating the contribution of *DIP2B* TREs to dystonic presentations.

### Diagnostic Benefit in Other Indications

ONT lrGS was also successful in achieving resolution of variant miscalling and missed variations, as well as in offering simultaneous genomic‐variant and episignature analysis. One individual, patient 11, was previously found by ES to have a potentially disease‐relevant^22^ *PI4KA* missense SNV, considered to be present in the homozygous state based on our ES‐pipeline output (Fig. S2). The associated recessive condition (neurodevelopmental disorder with spasticity, hypomyelinating leukodystrophy, and brain abnormalities; MIM: 616531) matched the patient's phenotype, consisting of dystonia‐spasticity, developmental delay, and cerebral hypomyelination. Because molecular analysis of *PI4KA* can be complicated by its pseudogenes *PI4KAP1* and *PI4KAP2*,^21^ we sought to further evaluate the homozygous variant call and characterized it as a false positive (Table 1; Fig. S2). When looking at ONT data covering the query region, the ES‐prioritized SNV occurred in ~50% of reads, consistent with a heterozygous alteration, and was accompanied by a nearby, previously undetected common polymorphism on the other allele. We assumed that the particular combination of the rare SNV with the polymorphism favored a mismapping of *PI4KA* parent gene‐derived reads onto the pseudogene *PI4KAP2*, resulting in the homozygous artifact variant; patient 11 remained currently undiagnosed. In another case (patient 12), a seemingly homozygous splice‐site SNV in *OTUD7A* was detected by ES, deemed to be possibly causative. However, segregation analysis identified the SNV heterozygously in only one parent, questioning the homozygosity in the patient.^35^ ONT lrGS additionally showed a heterozygous ~1.65‐Mb deletion encompassing *OTUD7A*, and manual phasing determined that the SNV and the SV were in *trans* (Table 1; Fig. S3A). This established the compound heterozygous diagnosis^22^, ^25^, ^26^ of a so far poorly delineated neurodevelopmental disorder (MIM: 620790) and expanded the associated clinical spectrum to include dystonia^36^ (Table 1). An updated bioinformatics investigation later demonstrated that the deletion was visible in patient 12's ES data but had not been called because of pipeline limitations,^1^, ^13^ underscoring the previously reported challenges in disentangling combinations of SNVs and SVs that overlap in *trans*.^13^ Moreover, we uncovered in patient 13 the missing molecular cause of a suspected clinical diagnosis; this female individual developed microcephaly and combined dystonia. On manual review of her ONT data, applying dedicated attention to variants affecting Rett/Rett‐like syndrome–related genes,^27^ we found a heterozygous pathogenic^22^ indel in *FOXG1* underlying a congenital variant of Rett syndrome (MIM: 613454). This indel was not found on (re‐)review of the patient's SRS data because there was no ES‐read coverage in the corresponding region of the gene, likely because of high GC content (Table 1; Fig. S3B). Lastly, in patient 14 with childhood‐onset dystonia, who was enrolled in our study after the SRS‐based detection of an in‐frame indel VUS^22^ in *KMT2B*, we probed the recently proposed utility of ONT lrGS to measure methylation levels at genomic positions that constitute an episignature.^7^ At 80 CpG sites selected for analysis, we observed a methylation pattern with high concordance to the specific microarray‐based episignature of *KMT2B*‐related dystonia^37^ (MIM: 617284; Table 1; Fig. S4A–C). This result together with the validation of the indel by ONT lrGS confirmed the diagnosis.^22^, ^37^

## Discussion

In this study, we performed lrGS enabled by ONT protocols to aid in diagnostic clarity for 14 individuals with dystonia who carried genomic alterations that were unclear from ES/srGS and/or impossible to resolve by SRS. The overall goal of our study was to investigate the merits of ONT lrGS in a “real‐world” scenario, facilitating future clinical implementation of this technology to obtain more accurate diagnoses and improve understanding of mutational mechanisms in the field of dystonia.^11^ Compared with other constitutional diseases where lrGS has begun to be more widely integrated,^8^, ^9^, ^10^, ^15^ this strategy has not yet seen relevant adoption in dystonia diagnostics.^11^ Our results now provide a number of key examples for the superior performance of long read–based variant discovery in heterogeneous dystonic presentations. First, we systematically used the method to follow up on unphased variants in cases for which other testing options had been exhausted, demonstrating successful long‐range read–based haplotype determination. Second, we observed marked improvements in the detection and resolution of variable SV events, rendering ONT lrGS a meaningful tool to complement or replace imperfect existing detection strategies for these mutations in dystonia.^11^ Third, we identified a distinctive advantage of ONT lrGS over SRS in the characterization of dystonia‐linked TREs, potentially justifying its incorporation as a first‐line screening test when repeat‐expansion pathologies are part of the differential diagnosis.^38^ Finally, our analysis of dystonic individuals with other difficult‐to‐map or uninterpretable variations highlighted additional accuracy gained in diagnostic evaluation by having access to the profiling of gene‐pseudogene pairs, comprehensive sets of different clinically important variant types, as well as haplotype‐aware DNA methylation. More broadly, our case examples suggest that caution should be practiced in daily settings of dystonia care when negative results are returned from routine genetic testing, such as ES.^2^, ^3^ Dystonia specialists should be aware that several genomic aberrations underlying their patients' conditions may currently remain ambiguous or go undetected because of technical difficulties in identifying them.^11^ Our findings reinforce the notion that both positive and negative ES/srGS results should be interpreted with scrutiny, especially in the context of SVs, TREs, and other alterations that require more advanced read‐alignment and variant‐calling methods.^6^, ^18^ We further conclude that current ES‐data pipelines and their standard bioinformatics software do not adequately account for the high level of genomic variation that can result in dystonia.^11^, ^13^ We found that the quality of causal variant calls was decisively enhanced in our ONT lrGS experiments. For example, ONT lrGS uniquely redefined the identity of different low‐confidence CNV calls from ES, including incorrectly proposed simple single‐exon deletions in *HIBCH* (patient 4) and *SGCE* (patient 7), as well as a potential *MECP2* cxSV with imprecise evidence (patient 5). For these three cases, we observed especially high levels of discordance between the outputs from ES and ONT lrGS; the latter platform yielded cxSVs involving deleted, duplicated, and inverted sequences (patients 4 and 5) and a LINE‐1 mobile‐element insertion (patient 7). Notably, mobile‐element discovery is often not a well‐established step in routine SRS data workflows, and available detection tools produce many false positives,^8^ hindering reliable diagnosis. Because there have not been many published studies on the contribution of mobile‐element insertions to movement disorders,^39^ we expect that their role in dystonia will be more precisely characterized when lrGS will be carried out in larger groups of affected individuals. As another illustration of the benefits of ONT lrGS over SRS, we stress a considerable degree of disagreement in the results from TRE genotyping; we were able to call dystonia‐associated repeat‐unit lengths more robustly by lrGS, improving curation of pathogenicity, and meticulous inspection of ONT reads indicated considerable variance in TRE sizes within individual subjects (eg, *FMR1*, patient 9), compatible with mosaicism.^15^ More comprehensive approaches using long‐read technology should be taken in the future to examine the clinical relevance of mosaicism in dystonia‐causing TRE disorders.^40^ We also highlight that systematic ONT‐based interrogation of genes associated with the clinically suspected diagnosis allowed for detection of a pathogenic indel that was missed by ES (*FOXG1*, patient 13). This is consistent with previous results showing that ONT lrGS may be more sensitive in calling small variants than SRS platforms.^8^ In addition, the successfully combined genome and methylome analysis highlights the utility of ONT lrGS for dystonic diseases in which methylation profiling and episignature studies are diagnostically important.^12^, ^37^

One caveat of our study is that we focused our evaluation on selected patients with mostly preidentified variant calls that were imprecise and inaccurate or with clinical findings that suggested a particular monogenic diagnosis. Our study was not designed to conduct a genome‐wide screening for causative variants by ONT lrGS in a larger group of molecularly undiagnosed patients. The diagnostic findings obtained in our present ONT lrGS analysis were validated or cross‐validated using different strategies, including simultaneous direct visualizations of annotated SRS and LRS data, follow‐up Sanger confirmation, quantitative polymerase chain reaction (PCR) or single‐nucleotide polymorphism array‐based methods, other PCR‐based techniques,^13^ and conventional episignature testing^37^ (Supporting Information Methods). To assess known existing ambiguity in dystonia‐related variation in a truly holistic manner, further studies involving unresolved patients with different forms of dystonia are required. For this purpose, a benchmarking of the accuracy of the ONT protocol in comparison with SRS‐derived outputs (ES/srGS) would be important. Unfortunately, we were unable to perform a direct comparative analysis for the dystonia samples because all patient SRS data were based on GRCh37/hg19 annotations, whereas the newly generated ONT data were annotated on GRCh38/hg38. Given that prior studies determined relevant discordances between GRCh37/hg19 and GRCh38/hg38 assemblies,^41^ highlighting the need for caution when “translating” genomic variants between the different assembly versions,^41^ it was not possible to confidently define methodological differences in sensitivity and specificity for genome‐wide variant identification between our ONT approach and SRS methods in dystonia. Nevertheless, we used an in‐house Illumina short‐read genome‐sequenced Genome‐in‐a‐Bottle (GIAB) sample (GRCh38/hg38)^42^, ^43^ and in‐house ONT sequencing data for a GIAB sample (GRCh38/hg38) to compare the number of small variants (SNVs/indels) identified by each sequencing technology (Fig. S5; Table S1): we found that 95.6% of quality‐controlled SNVs/indels were concordantly called by srGS and ONT lrGS. Similar results were previously reported by Negi and colleagues,^44^ showing high concordance between functional annotated SNVs detected by SRS and LRS of >90%. Thus, despite a reported lower per‐base accuracy,^6^ ONT lrGS may be considered a valid diagnostic strategy with future clinical value because the method detects a diversity of variant types while SRS‐identified SNV/indel calls are largely covered. In this context, further research should also explore the performance of other LRS platforms such as PacBio HiFi sequencing in the diagnosis of dystonia^11^; PacBio‐based sequencing offers higher accuracy but lower throughput,^6^, ^11^ and it remains to be seen whether the advantages of ONT analysis in turnaround time and processing scale will drive its implementation in clinical diagnostics of dystonia, or whether the methods can be used interchangeably or in a complementary fashion. To further increase the diagnostic yield, future studies using other advanced molecular‐profiling strategies such as multidimensional omics analyses may be useful.

In summary, we took a first step toward describing the diagnostic value of ONT lrGS in dystonia, addressing the imprecision of current standard SRS pipelines in diagnosing cases related to more challenging variation. Our data emphasize the need for additional studies into the advantage of the method in driving precision diagnosis and mechanistic elucidation in dystonia.

## Author Roles

Conceptualization, U.S. and M.Z.; Data curation, U.S., M.P., E. Tilch, A.S., S.L., S.E., E.G., and M.Z.; Investigation, U.S., M.P., N.M.‐S., M.B., T.B., E. Tsoma, I.D., P. Harrer, A.M.S., M.W., B.W., S.A., A.K., A.D.F., M.K., K.K., P. Havrankova, R.T., S.L., J.N., M.S., E.G., and M.Z.; Software, M.P., E. Tilch, A.S., and S.E.; Writing—original draft preparation, U.S. and M.Z.; Writing—review and editing, H.P., K.O., and M.Z.; Visualization, U.S. and M.Z.; Supervision, R.J., S.B., J.W., and M.Z.; Project administration, J.W. and M.Z. All authors read and approved the final manuscript.

## Financial Disclosures of All Authors (for the Past 12 Months)

The authors report no disclosures.

## Supporting information


**Data S1.** Supporting Figures.

## Data Availability

The data that support the findings of this study are available on request from the corresponding author. The data are not publicly available due to privacy or ethical restrictions.
