# Supplementary material for: Integrating Long‐Read Nanopore Sequencing for Precision Resolution of Genomic Variants in Dystonia
Source: Mov Disord. 2025 Sep 30;41(1):70–83. doi: 10.1002/mds.70072 (PMC12882031; doi:10.1002/mds.70072)
Supplement: Supplementary file 1 — Data S1. Supporting Figures. [file MDS-41-70-s001.pdf]

SUPPLEMENTARY MATERIAL

Table of contents

Supplementary Methods

Supplementary Figures

Supplementary Figure 1
Supplementary Figure 2
Supplementary Figure 3
Supplementary Figure 4
Supplementary Figure 5
Supplementary Figure 6

Supplementary Table

Supplementary References

Supplementary Methods

Variant analysis in ONT data

The following packages were deployed with default parameters for the study of different variant types and DNA-base modifications in aligned ONT lrGS BAM files: the diploid variant calling program Clair3 v1.0.4 (https://github.com/HKU-BAL/Clair3) for detection of SNVs and indels; Spectre v0.2 (https://github.com/fritzsedlazeck/Spectre) for identification of CNVs; Sniffles2 v2.4 (https://github.com/fritzsedlazeck/Sniffles) for discovery of other SVs including cxSVs; straglr v1.4.5 (https://github.com/bcgsc/straglr) for TRE genotyping; and modkit v0.4 (https://github.com/nanoporetech/modkit) for scrutiny of CpG methylation marks in selected samples for which assaying CpG methylation was considered useful for data interpretation. We called variants of interest by the respective software and confirmed disease-causing alterations visually by using the Integrative Genome Viewer (IGV)[1]. Variants were phased by implementing the haplotype phasing tool "Whatshap" (https://whatshap.readthedocs.io/en/latest/) and by selecting the "Group alignments by: phase" option in IGV. Coordinates of SV breakpoints were determined by visual inspection of read alignments and human BLAT tool searches. TRE analysis was carried out at the locus of interest and repeat patterns were visually assessed based on the aligned reads using IGV. The missing variants in two patients were identified by searching the variant-output files provided by Clair3 and Spectre for mutational changes of genes associated with the suspected clinical differential diagnosis. Methylation profiles were highlighted in IGV by coloring alignments by modified bases. In the context of a *KMT2B* variant, we evaluated methylation information at 80 CpG sites that had been identified to contribute to the definition of a *KMT2B*-related dystonia-specific episignature through previous microarray experiments[2]. More specifically, methylation data used in the downstream analysis were obtained from aligned ONT lrGS BAM files with the help of modkit. We followed recently published principles and recommended approaches to determine the similarity between ONT lrGS- and array-based episignatures for mutant *KMT2B* (see reference[3]). Methylation data were extracted from ONT data at pre-defined episignature CpG sites (n=80, see Suppl.Fig.4) using GRCh38/hg38 coordinates. Hierarchical clustering analysis and support-vector machine (SVM) development were undertaken, as previously reported[2-5]. Following SVM training, SVM-based analysis was performed to predict the presence of a *KMT2B*-specific episignature in patient-14, considering 37 control-subject samples and four samples from dystonia-affected individuals without *KMT2B* rare variants as reference.

Variant confirmation was carried out as appropriate. All variations were visually confirmed by direct parallel manual assessments in annotated SRS and ONT lrGS data. Exact breakpoint locations of each SV were determined based on IGV visualizations[1]. SNV/indel and simple small CNV events were confirmed with Sanger sequencing. Larger SVs/cxSVs and TREs were validated using combinations of qPCR, SNP array-based methods, or other PCR-based techniques including conventional amplification of breakpoints with follow-up Sanger analysis and/or fragment length analysis[6-8]. Conventional *KMT2B* episignature testing was also performed for validation[2]. Validations of SVs and TREs were carried out in external accredited laboratories[8].

Supplementary Figures

Suppl. Figure 1 Successful variant phasing performed on patient-3

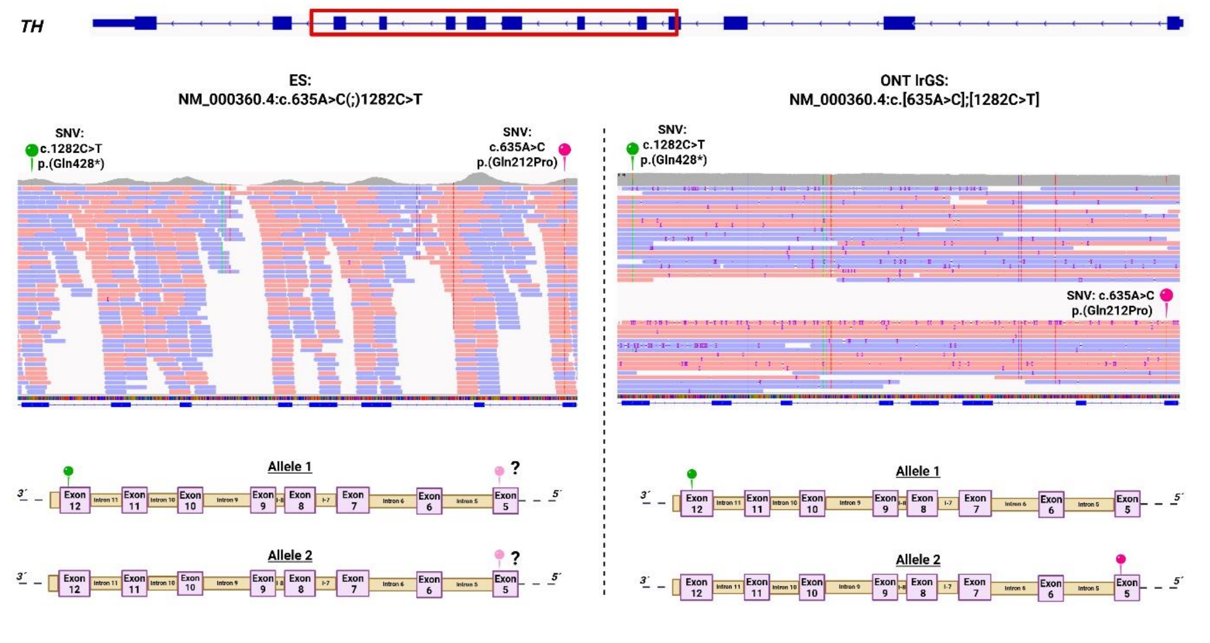

Visualizations of genomic alignments of previously acquired ES data[6, 8] and herein-produced ONT lrGS data in the IGV software[1] are shown. ES variant annotations were based on genome assembly GRCh37/hg19[6, 8], while ONT lrGS data were analyzed using GRCh38/hg38. Relevant parts of the canonical transcript of the analyzed gene are illustrated at the top of the comparison between ES and ONT lrGS outcomes. IGV zoom-in regions highlight the called variants, which are described according to HGVS nomenclature. Schematic representations of the allelic configurations before and after resolution by LRS are depicted. Alignments are phased into separate haplotypes to determine that the two variants were in *trans*, establishing a compound heterozygous diagnosis related to *TH* in patient-3. ES, exome sequencing; HGVS, Human Genome Variation Society; IGV, Integrative Genomics Viewer; LRS, long-read sequencing; ONT lrGS, Oxford Nanopore Technologies-based long-read genome sequencing; SNV, single-nucleotide variant.

Suppl. Figure 2 Resolution of pseudogene-associated read misalignment with a false-positive variant call in patient-11

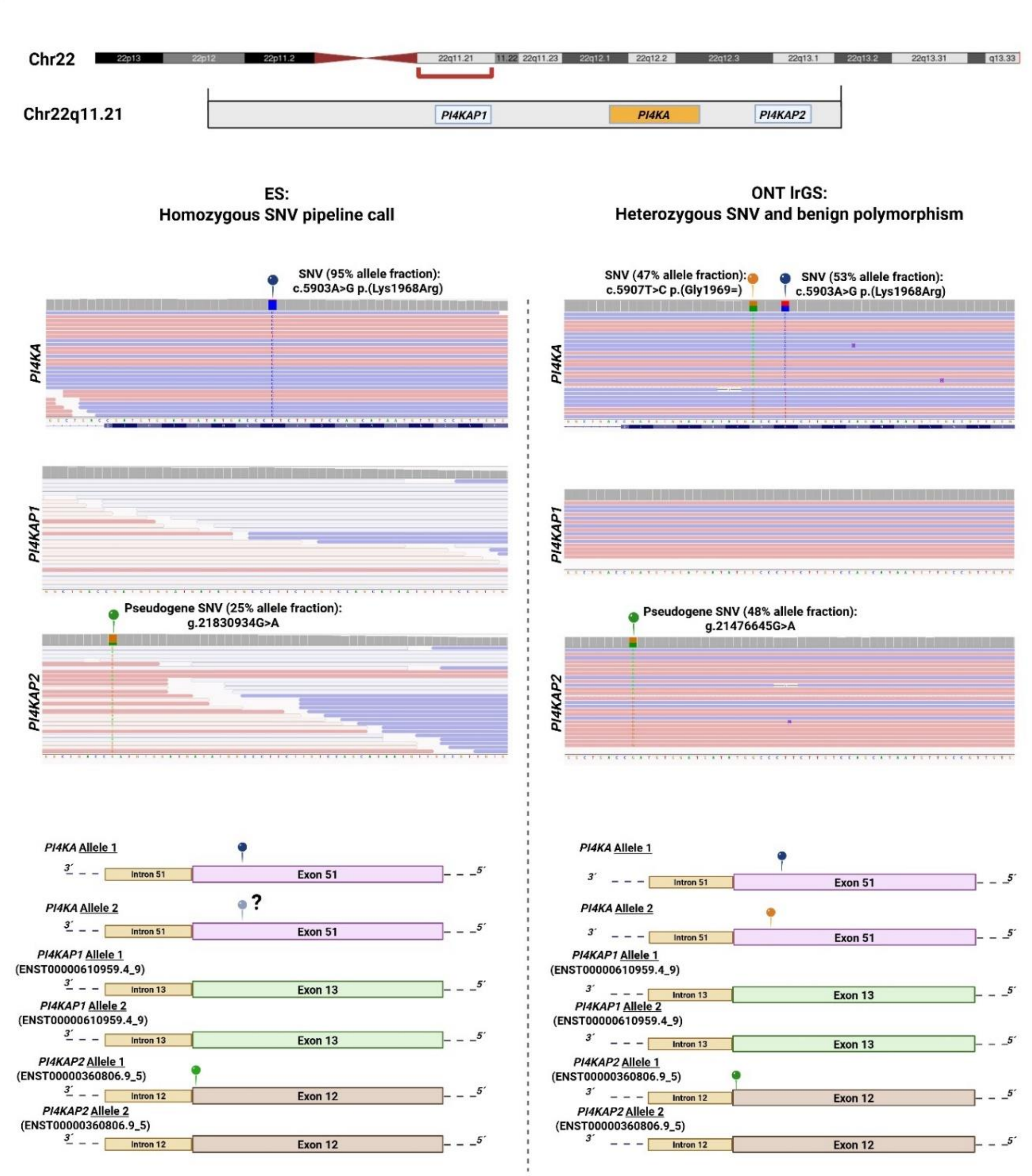

Visualizations of genomic alignments of previously acquired ES data[6, 8] and herein-produced ONT lrGS data in the IGV software[1] are shown. ES variant annotations were based on genome assembly GRCh37/hg19[6, 8], while coordinates provided for ONT lrGS data refer to GRCh38/hg38. A chromosomal ideogram of the 22q11.21 region containing *PI4KA* and its two pseudogenes *PI4KAP1* and *PI4KAP2* is illustrated at the top of the comparison between ES and ONT lrGS outcomes. IGV zoom-in regions highlight the called variants, along with their read-based variant allele fractions. Schematic representations of the allelic configurations before and after resolution by LRS are depicted. Misalignment of *PI4KA* reads caused by a rare missense substitution in combination with a synonymous polymorphism resulted in a false-positive homozygous variant call in the ES pipeline, which was initially considered a disease-related candidate for patient-11. ONT lrGS mapped the reads to the correct positions and invalidated the homozygous candidate *PI4KA* alteration. ES, exome sequencing; IGV, Integrative Genomics Viewer; LRS, long-read sequencing; ONT lrGS, Oxford Nanopore Technologies-based long-read genome sequencing; SNV, single-nucleotide variant.

Suppl. Figure 3 Missing variants in patients 12 and 13 identified by direct inspection of LRS data

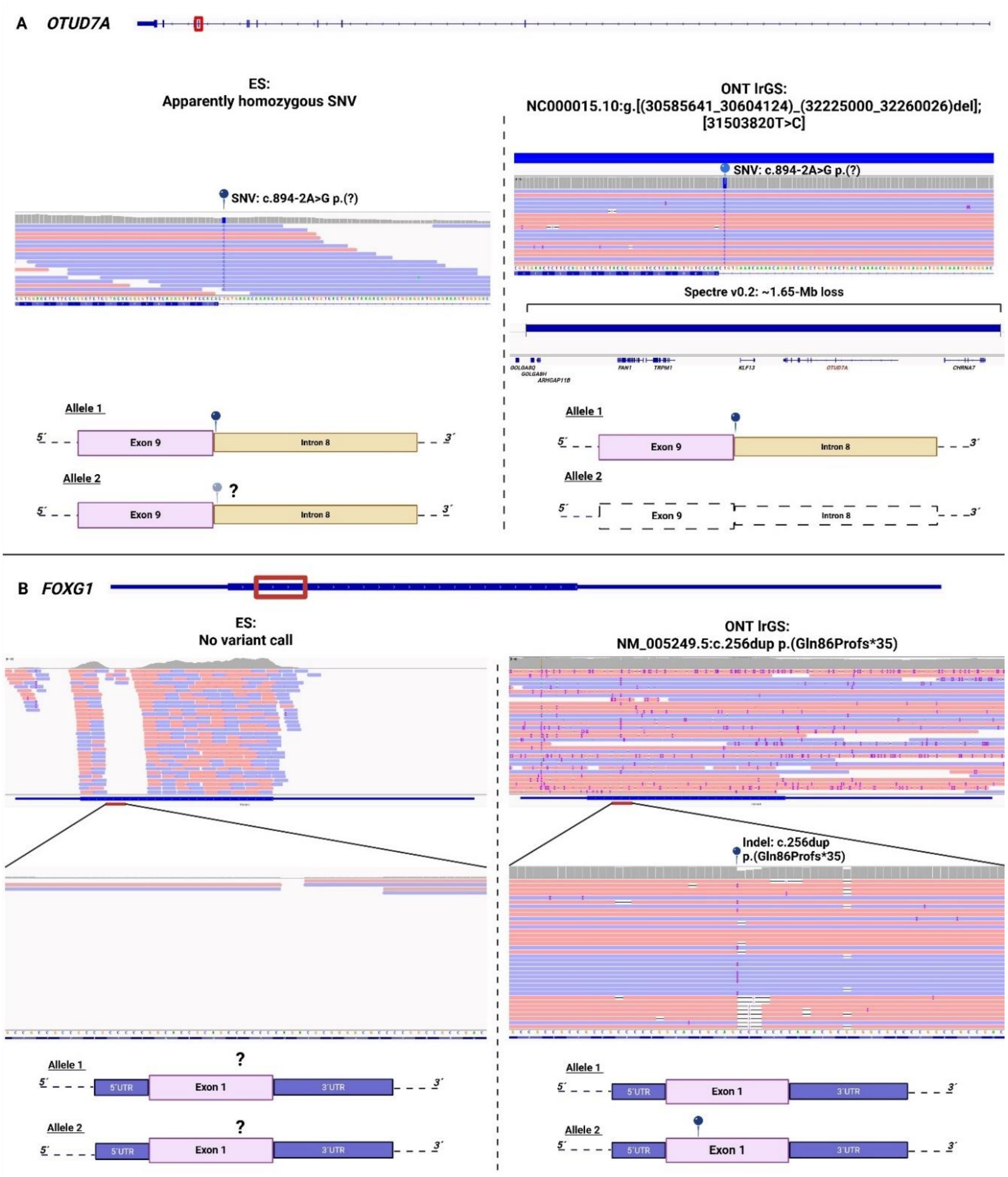

Visualizations of genomic alignments of previously acquired ES data[6, 8] and herein-produced ONT lrGS data in the IGV software[1] are shown. ES variant annotations were based on genome assembly GRCh37/hg19[6, 8], while coordinates provided for ONT lrGS data refer to GRCh38/hg38. The transcript (or relevant parts of the transcript) of each analyzed gene are illustrated at the top of each comparison between ES and ONT lrGS outcomes. IGV zoom-in regions highlight the positions with missed variants and the called variants; variants are described according to HGVS nomenclature. Schematic representations of the allelic configurations before and after resolution by LRS are depicted. (A) In patient-12, analysis of ONT lrGS data with different variant callers uncovered a previously undetected larger deletion containing the entire coding sequence of *OTUD7A* in addition to an *OTUD7A* SNV, completing the diagnosis of a rare autosomal recessive neurodevelopmental disorder[9]. (B) In patient-13, ONT lrGS identified a *FOXG1* indel in a GC-rich region without coverage in ES data. ES, exome sequencing; HGVS, Human Genome Variation Society; IGV, Integrative Genomics Viewer; indel, short insertion/deletion; LRS, long-read sequencing; ONT lrGS, Oxford Nanopore Technologies-based long-read genome sequencing; SNV, single-nucleotide variant.

Suppl. Figure 4 Parallel detection of a rare variant in *KMT2B* and methylation at 80 episignature-defining CpG sites in patient-14

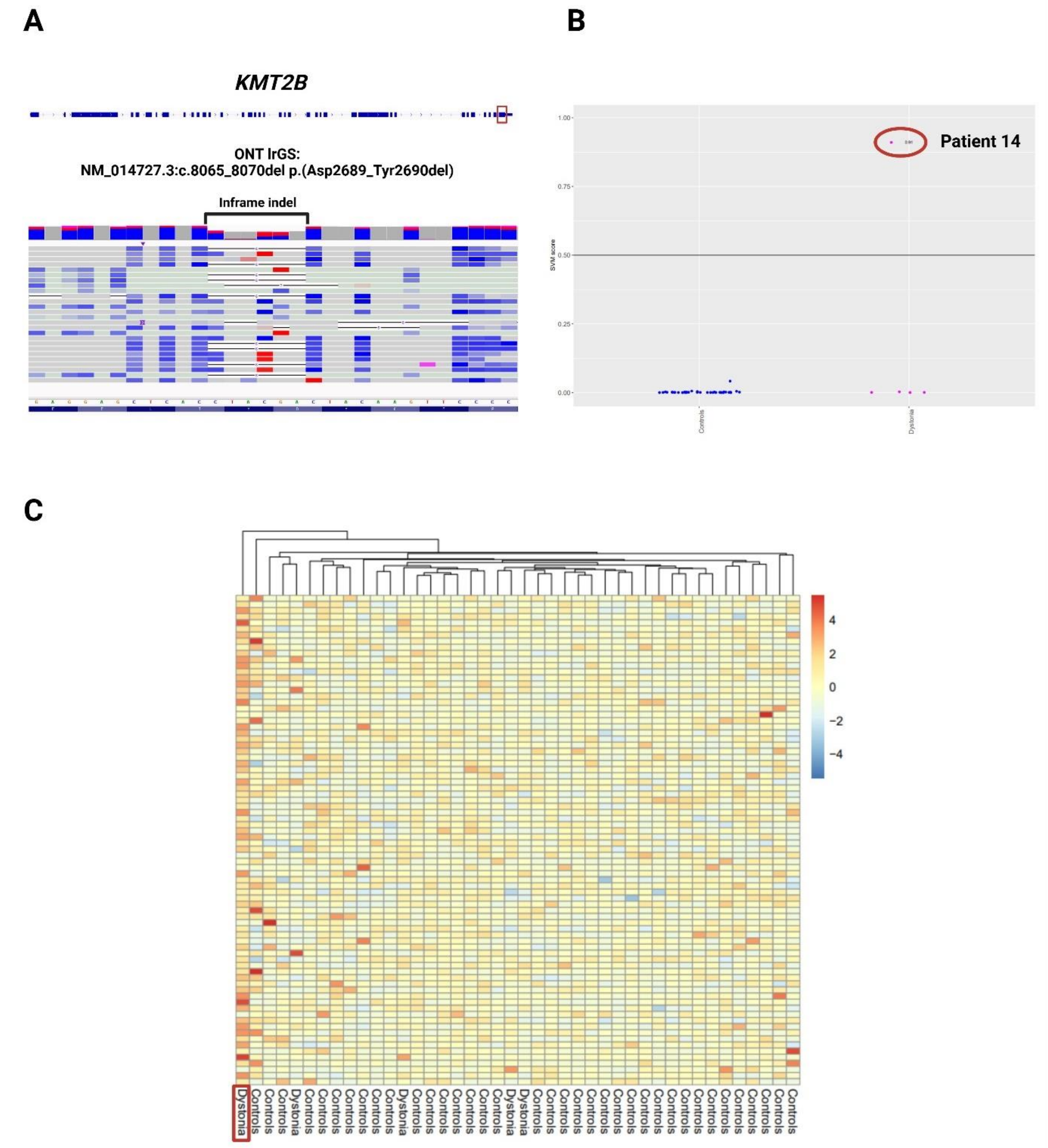

(A) Visualization of genomic alignments of ONT lrGS data in the IGV software[1] is shown. The IGV zoom-in panel highlights the called *KMT2B* indel variant, originally classified as a VUS[10], in patient-14. (B) ONT lrGS-based methylation analysis allowed to distinguish the sample with the *KMT2B* indel from controls (SVM score of 0.91; a score >0.5 indicates that a sample is more likely to bear a disease-related *KMT2B* alteration than not to bear such mutation). ONT lrGS data from 37 control subjects and four dystonia-affected individuals without rare non-synonymous *KMT2B* variants were included in the analysis. The *KMT2B* VUS was subsequently re-classified as likely pathogenic. Methylation patterns at 80 CpG sites that were previously identified to contribute to the definition of a *KMT2B* dystonia-typical episignature[2] were evaluated. (C) Results from hierarchical clustering analysis based on ONT lrGS data-derived methylation levels (patient-14 with *KMT2B* VUS, 37 control subjects, 4 dystonia-affected individuals without rare non-synonymous *KMT2B* variants). IGV, Integrative Genomics Viewer; indel, short insertion/deletion; ONT lrGS, Oxford Nanopore Technologies-based long-read genome sequencing; SVM, support vector machine; VUS, variant of uncertain significance.

Suppl. Figure 5 Comparison of SNV/indel calls (variations < 50bp) between srGS and ONT lrGS

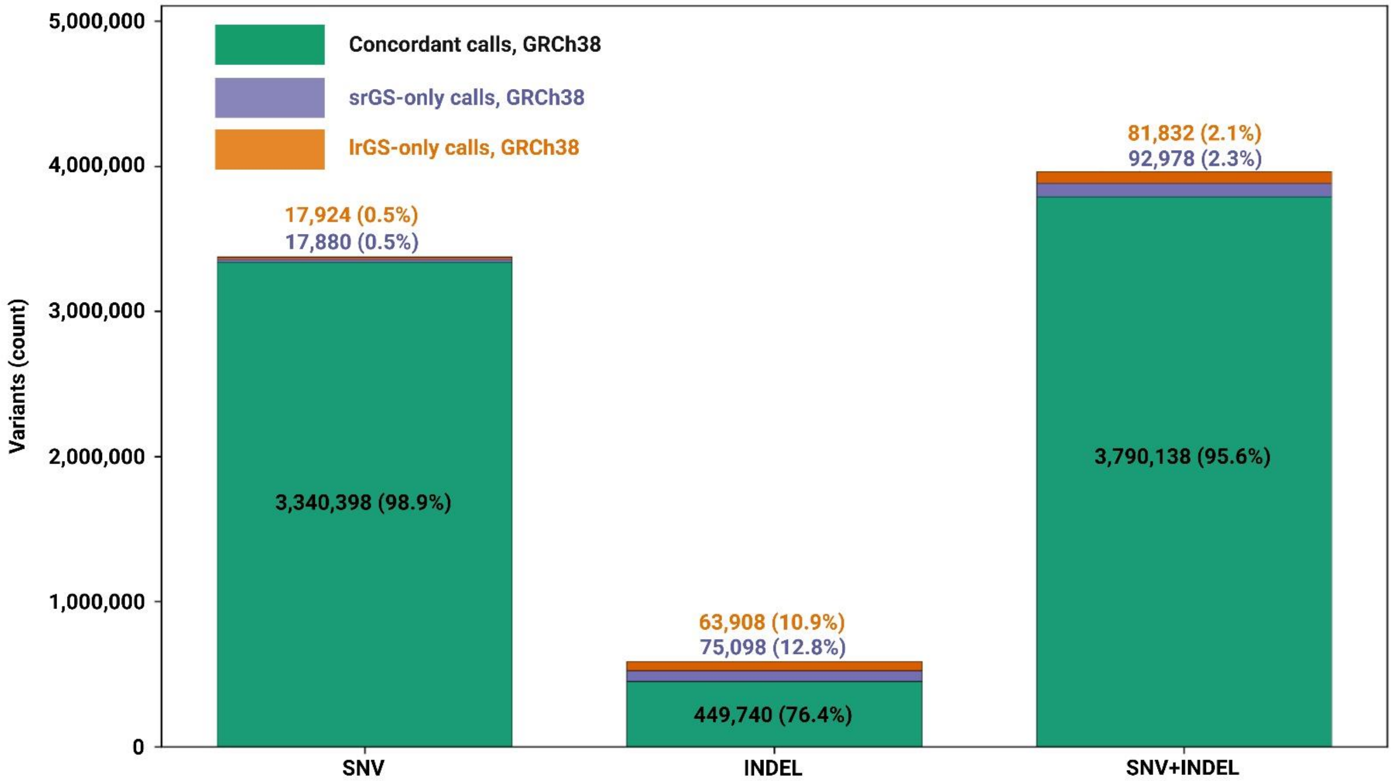

Breakdown of variant calls (SNVs and indels) that were condordant between srGS (Illumina) and ONT lrGS, or identified by srGS only or by ONT lrGS only. The analysis was conducted with in-house sequencing data produced for a Genome in a Bottle (GIAB) sample[11], annotated on GRCh38/hg38, and focused on variants within GIAB`s high confidence regions that passed the quality control filter. Indel, short insertion/deletion; ONT lrGS, Oxford Nanopore Technologies-based long-read genome sequencing; SNV, single-nucleotide variant; srGS, short-read genome sequencing.

Suppl. Figure 6 Sorted reads carrying *FMR1* TREs of variable size in patient-9

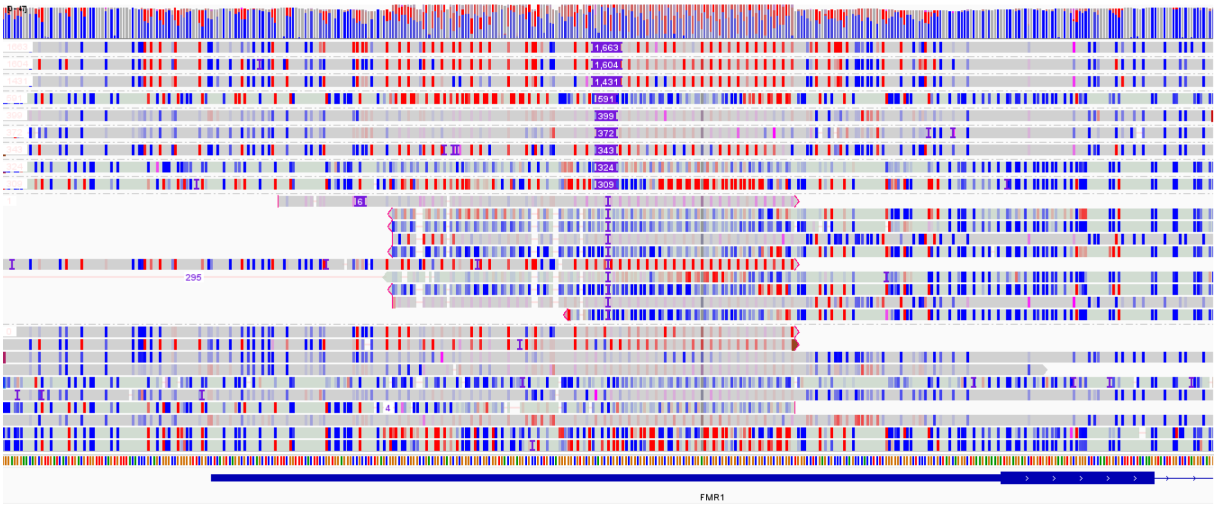

The suspected mosaic constellation of expanded repeat units is highlighted. *FMR1* methylation is marked in red in the ONT data. Note hypermethylation of long expanded repeats (first four reads). ONT lrGS, Oxford Nanopore Technologies-based long-read genome sequencing; TRE, tandem repeat expansion.

Supplementary Table

Suppl. Table Summary of variant calls (SNVs and indels, < 50bp) produced for a Genome in a Bottle (GIAB) sample[11] by srGS and ONT lrGS

Variant type	Total calls (srGS)	Total calls (ONT lrGS)	Concordant calls (srGS-ONT lrGS)	srGS-only calls	ONT lrGS-only calls	Union (srGS, ONT lrGS calls)	Concordance (concordant calls/union)	srGS discordance (srGS-only calls/union)	ONT lrGS discordance (ONT lrGS-only calls/union)
**SNV**	3358278	3358322	3340398	17880	17924	3376202	0.989395184	0.005295892	0.005308924
**INDEL**	524838	513648	449740	75098	63908	588746	0.763894787	0.127555856	0.108549357
**SNV+INDEL**	3883116	3871970	3790138	92978	81832	3964948	0.955911149	0.023449992	0.020638858

The analysis focused on variants within GIAB`s high confidence regions that passed the quality control filter. Union = concordant calls (srGS-ONT lrGS) + srGS-only calls + ONT lrGS-only calls.
Indel, short insertion/deletion; ONT lrGS, Oxford Nanopore Technologies-based long-read genome sequencing; SNV, single-nucleotide variant; srGS, short-read genome sequencing.

Supplementary References

1. Robinson JT, Thorvaldsdottir H, Winckler W, et al. Integrative genomics viewer. Nat Biotechnol 2011;29(1):24-26.
2. Mirza-Schreiber N, Zech M, Wilson R, et al. Blood DNA methylation provides an accurate biomarker of KMT2B-related dystonia and predicts onset. Brain 2021.
3. Geysens M, Huremagic B, Souche E, et al. Clinical evaluation of long-read sequencing-based episignature detection in developmental disorders. Genome Med 2025;17(1):1.
4. Oexle K, Zech M, Stuhn LG, et al. Episignature analysis of moderate effects and mosaics. Eur J Hum Genet 2023;31(9):1032-1039.
5. Stehr AM, Fischer J, Mirza-Schreiber N, et al. Variable expressivity of KMT2B variants at codon 2565 in patients with dystonia and developmental disorders. Parkinsonism Relat Disord 2025;133:107319.
6. Zech M, Jech R, Boesch S, et al. Monogenic variants in dystonia: an exome-wide sequencing study. Lancet Neurol 2020;19(11):908-918.
7. Dzinovic I, Boesch S, Skorvanek M, et al. Genetic overlap between dystonia and other neurologic disorders: A study of 1,100 exomes. Parkinsonism Relat Disord 2022;102:1-6.
8. Zech M, Dzinovic I, Skorvanek M, et al. Combined genomics and proteomics unveils elusive variants and vast aetiologic heterogeneity in dystonia. Brain 2025.
9. Suzuki H, Inaba M, Yamada M, et al. Biallelic loss of OTUD7A causes severe muscular hypotonia, intellectual disability, and seizures. Am J Med Genet A 2021;185(4):1182-1186.
10. Richards S, Aziz N, Bale S, et al. Standards and guidelines for the interpretation of sequence variants: a joint consensus recommendation of the American College of Medical Genetics and Genomics and the Association for Molecular Pathology. Genet Med 2015;17(5):405-424.
11. Zook JM, McDaniel J, Olson ND, et al. An open resource for accurately benchmarking small variant and reference calls. Nat Biotechnol 2019;37(5):561-566.
